# A framework for categorizing electrode montages in transcranial direct current stimulation

**DOI:** 10.3389/fnhum.2015.00054

**Published:** 2015-02-06

**Authors:** Padideh Nasseri, Michael A. Nitsche, Hamed Ekhtiari

**Affiliations:** ^1^Neurocognitive Laboratory, Iranian National Center for Addiction Studies, Tehran University of Medical SciencesTehran, Iran; ^2^Translational Neuroscience Program, Iranian Institute for Cognitive Sciences Studies (ICSS)Tehran, Iran; ^3^Department of Clinical Neurophysiology, University Medical Center, Georg-August-Universität GöttingenGöttingen, Germany

**Keywords:** transcranial electrical stimulation (TES), transcranial direct current stimulation (tDCS), non-invasive brain stimulation, multi-electrode stimulation, electrode montages

Transcranial Direct Current Stimulation (tDCS) is a non-invasive brain stimulation technique that has been reintroduced in the last decade and is now mainly used as a cognitive modulator in human neuroscience research. tDCS delivers a weak direct current (usually up to 2 mA) over the scalp and creates a constant electric field in the brain which can lead to acute alterations of the excitability of cortical areas by its subthreshold depolarizing or hyperpolarizing effects on neuronal resting membrane potentials (Nitsche and Paulus, 2000). Beyond these acute effects, stimulation for some minutes results in neuroplastic after-effects, which can last for over 1 h after stimulation (Nitsche and Paulus, 2001). With repeated usage, longer lasting effects can be induced, which are in the range of late-phase plasticity (Monte-Silva et al., 2013). The neuroplastic effects resemble LTP- and LTD-like plasticity of glutamatergic synapses (Liebetanz et al., 2002; Nitsche et al., 2003a). Therefore, this technique allows us to study neuroplasticity of the human brain in a reversible manner and to modulate plasticity-related functions such as memory or learning, which critically depend on neuroplasticity, in healthy and clinical populations.

Traditionally, one or more surface-positive (anode) and negative (cathode) electrodes are used to deliver current; one is positioned over the target area and the other one is put over another cranial (intracephalic) or extracranial (extracephalic) region of the body. These electrodes are usually called active and reference electrode respectively. However, these terms can be technically improper and should be replaced with other terms such as “target” and “return” electrodes, because the size and the place of a return electrode have an impact on its effects and thus it might not be physiologically inert. The return electrode can contribute directly—and not only via determination of electrical field orientation—to physiological effects when put over the cranium as well (Brunoni et al., 2012). Several studies have also shown antagonistic effects of stimulation on visual cortex (Antal et al., 2004; Accornero et al., 2007) and motor cortex (Nitsche and Paulus, 2000) dependent on return electrode position. In any case, the position of the return electrode will affect electrical field orientation, which is critical for the efficacy, and direction of the effects (Bikson et al., 2010; Kabakov et al., 2012). In both—extracephalic and intracephalic conditions—positive (cathode) and negative (anode) poles are conventionally physiologically distinguished according to their effects on excitability of the brain. Basically, cathodal stimulation has hyperpolarizing effects, which lead to inhibition of cortical activity, while anodal stimulation has excitatory effects (Nitsche et al., 2003b, 2008). It should be worth noting that although every neuron undergoes hyperpolarizing and depolarizing, the physiological effect depends more on axonal/soma polarization (Arlotti et al., 2012), hence the physical and physiological aspects can be dissociated. General effects on excitability, which were obtained primarily in the human motor cortex, might also switch, turning from excitatory to inhibitory or vice versa, dependent on stimulation parameters such as intensity, and duration (Batsikadze et al., 2013; Monte-Silva et al., 2013), and position of the return electrode (Antal et al., 2004; Accornero et al., 2007). With a rise in prevalence of studies using tDCS, protocols have become more complex and varieties of tDCS montages were introduced and are used in different labs. Despite this extending diversity of tDCS electrode montages, to our best knowledge, there is no consensus among researchers in this field on a systematic framework for categorizing electrode montages in a unified way. In this short article, we propose a framework for categorization of tDCS montages according to physical characteristics. This categorization is based on published studies until October 2014. Our main motivation to propose this framework is to unify the classifications of electrode montages in a simple way; there are nevertheless several other advantages of this categorization. First, different montages that are used to target a specific brain area such as dorsolateral prefrontal cortex (DLPFC) could have different effects; therefore providing a unified classification enables us to take these differences into account. Furthermore, this classification gives us a chance to explore other novel potentials for electrode montages that so far have remained untouched. Lastly, a unified systematic framework will be helpful for presenting study methods and for extracting data for systematic reviews and meta-analyses in a more practical way.

## Categorization of tDCS montages

Based on affected hemispheres and the number of electrodes, we categorized montages for conventional electrodes in 4 groups and 12 subgroups. We have used the 10/20 EEG system to localize areas on the cranium. For describing electrode montages, we state the position of the anode electrodes first. For example, F3/F4 refers to the anodal electrode over the F3 and cathodal electrode over the F4 region.

### Unilateral

In this group, only one hemisphere is targeted for stimulation. It can be divided into 3 subgroups:

#### Monopolar

**Figure 1:**
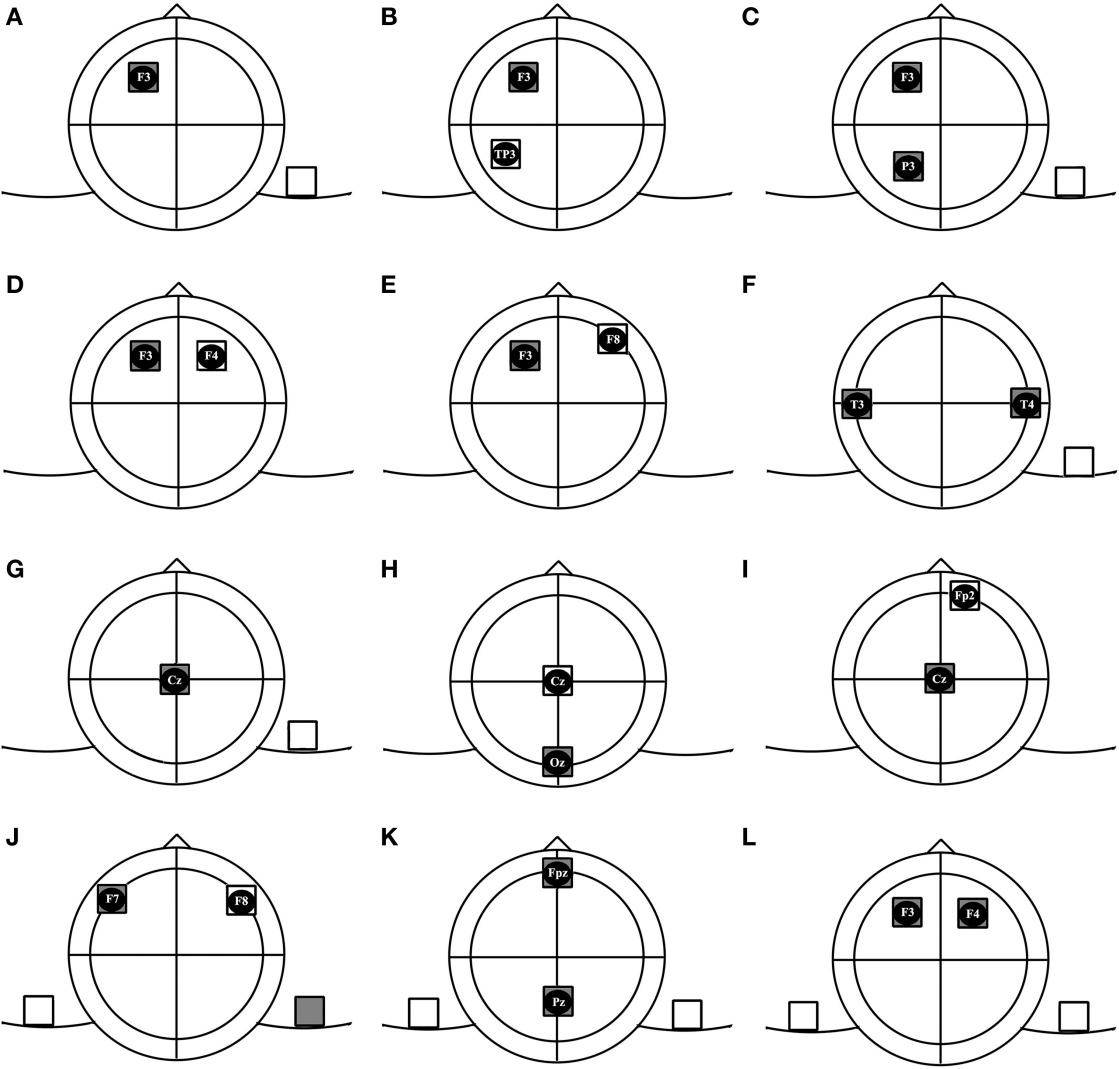
**Subgroups of tDCS montages: (A)** unilateral monopolar, **(B)** unilateral bipolar, **(C)** unilateral multiple monopolar, **(D)** bilateral bipolar-balanced, **(E)** bilateral bipolar-non balanced, **(F)** bilateral multiple monopolar, **(G)** midline monopolar, **(H)** midline bipolar-balanced, **(I)** midline bipolar-non balanced, **(J)** dual channel- bipolar, **(K)** dual channel midline double monopolar, **(L)** dual channel bilateral double monopolar.

This term refers to positioning only one electric pole over the cranium. In this montage, one electrode is positioned on the scalp and the other one is placed on any other part of the body. As shown in Figure 1, an example of this montage is F3/contralateral shoulder (Fertonani et al., 2010).

#### Bipolar

In bipolar arrangements, both electric poles are placed over the brain. In the unilateral bipolar subgroup, both electrodes are positioned over the same hemisphere (e.g., F3/TP3). In this condition the targeted hemisphere is modulated while the other hemisphere is supposed to remain unaffected by direct effects of stimulation. Brunelin et al. (2012) used this montage to modulate auditory hallucinations in schizophrenic patients (Brunelin et al., 2012).

#### Multiple-monopolar

At least 3 electrodes are used in this montage. The target electrodes of identical polarity are placed over one hemisphere and the return electrode is positioned over another part of the body. We could not find any study incorporating this montage, but an example could be F3 and P3/contralateral shoulder, to modulate frontoparietal networks contributing in attention processing.

### Bilateral

Electrodes are placed bilaterally and both hemispheres are supposed to be affected by electric current.

#### Bipolar-balanced

The electrodes are placed symmetrically. This montage is supposed to be suitable for simultaneously activating a brain region and inhibiting its contralateral counterpart. An example of this montage is F3/F4, which is usually used to enhance excitability of the left DLPFC and to reduce excitability of the right DLPFC (Brunoni et al., 2013; Nelson et al., 2014).

#### Bipolar-nonbalanced

Same as the previous condition, the electrodes are positioned bilaterally, but are placed over different regions not symmetrically. For example, the anode could be placed over P3 and cathode over P6, which was Jacobson's montage of choice in his study on episodic memory (Jacobson et al., 2012).

#### Multiple-monopolar

Similar to the electrode arrangement in previous group, at least 3 electrodes are used in this montage. 2 target electrodes of the same polarity are placed bilaterally over two hemispheres and the third electrode (return) is put over any part of the body. An example is the T3andT4/right deltoid muscle montage that was used in some protocols for enhancing visual memory (Boggio et al., 2012; Lapenta et al., 2012).

### Midline

In this condition, the target electrode/s will be placed over the midline (e.g., Cz or Oz or Fz). These montages can be categorized in 3 subgroups:

#### Monopolar

In this type, the target electrode is placed over the midline area and the return electrode is placed over an extracephalic position (e.g., Fz/Left cheek to modulate inhibitory control) (Hsu et al., 2011).

#### Bipolar-balanced

Both electrodes will be placed over midline regions. One prevalent montage of this subgroup is Oz/Cz which is common in visual studies (Antal et al., 2001; Peters et al., 2013).

#### Bipolar-nonbalanced

The target electrode will be placed over the midline region with an intracephalic return electrode positioned over any part of scalp except midline. An example could be Cz/FP2 (Stagg et al., 2009).

### Dual channel

In this group, 2 pairs of electrodes are used, which are connected to two independent devices.

#### Bipolar

Two target electrodes with different polarities are placed symmetrically over the scalp. The return electrodes are put over ipsilateral parts of body. An example would be F7/left shoulder and right shoulder/F8. This montage provides us with an opportunity to perform anodal tDCS over F7 and cathodal tDCS over F8 simultaneously (Lee et al., 2013). The advantage of this montage compared to the classic Bilateral Bipolar Balanced montage, F7/F8, might be that this montage allows to produce distinct electrical fields in homolog regions of the brain with different intensity and timing, which might enable us to modify brain functions in a more specific manner. It should however be noted that this montage, as compared to the bilateral balanced montage leads to different current flow directions, which might result in different physiological effects. Hence, computational modeling for a better understanding of current flow patterns will be needed.

#### Midline double-monopolar

The montage is similar to the previous condition, but active electrodes are positioned over midline regions. An example is Fpz/right shoulder and Pz/left shoulder. To our best knowledge, no published study has ever used this type of montage.

#### Bilateral double-monopolar

Two electrodes of same polarity are placed over the scalp and 2 other electrodes will be positioned over the contralateral orbits or above contralateral parts of the body (e.g., P3/contralateral orbit and P4/contralateral orbit) (Klein et al., 2013).

## Functional considerations

The framework we are proposing is mainly based on physical electrode arrangements, but there are a few other points regarding tDCS montages that should be addressed. First, published articles in most cases consider montages with return electrodes above supraorbital regions as unilateral and monopolar, but supraorbital regions are situated over the frontal poles and orbitofrontal cortices and therefore these electrodes have an effect on brain functions (Kincses et al., 2003), unless large electrodes, which are functionally ineffective (Nitsche et al., 2007) are used. Thus, in most cases these arrangements resemble bipolar bilateral electrode montages. Hence, for the sake of physical characteristics and also functional efficacy, we suggest to classify these montages as bipolar.

Another important aspect is the size of electrodes. In some studies, electrodes are positioned bilaterally, but the size of the return electrode is enhanced to reduce the amount of current density, hence the functional efficacy of the return electrode might be eliminated (Nitsche et al., 2007) and therefore these montages have a function similar to monopolar montages. HD electrodes arrangements are also dissimilar in functional efficacy and physical configuration aspects. We haven't excluded these montages from their physical subgroups, but these differences should be taken into consideration in analyses of functional effects.

Furthermore, some montages such as Unilateral Multiple—Monopolar and Dual Channel montages can be used to stimulate brain networks rather than a region (Ruffini et al., 2014). This opportunity might allow us to investigate functional connectivity among brain regions and to modulate our targeted cognitive function more effectively. In these cases, the terms “target” and “return” electrodes might not be appropriate.

Finally, the main technical limitation in electrode positioning over the skull is the distance between edges of anodal and cathodal electrodes. Some modeling studies suggest keeping the edges of the electrodes at least 4 cm away from each other to reduce current shunting over the cranium (Moliadze et al., 2010). New computational models of brain current flow during tDCS are required to provide more accurate insights into real current flow patterns of these 12 groups of montages in the normal range of human cranium and brain.

The framework we have proposed is mainly based on physical arrangements, therefore some characteristics which have been shown to be important for physiological and functional effects (e.g., electrode distance, current intensity, stimulation duration), are not included. We hope that nevertheless this physical classification can create a common ground for researchers, facilitate communication, and will help to classify methods and approaches. This classification can be easily extended and modified in future studies for other transcranial electrical stimulation (tES) methods without anode vs. cathode electrodes such as transcranial alternating current stimulation (tACS) and transcranial random noise stimulation (tRNS) techniques and hopefully will help also in these cases to unify nomenclature, and to explore the parameter space systematically.

### Conflict of interest statement

The authors declare that the research was conducted in the absence of any commercial or financial relationships that could be construed as a potential conflict of interest.
